# 3,4-Dihalo-5-hydroxy-2(5*H*)-furanones: Highly Reactive Small Molecules

**DOI:** 10.3390/molecules29215149

**Published:** 2024-10-31

**Authors:** Katarzyna Żurawska, Anna Byczek-Wyrostek, Anna Kasprzycka, Krzysztof Walczak

**Affiliations:** 1 Centre of Biotechnology, Silesian University of Technology, Krzywoustego 8, 44-100 Gliwice, Poland; katarzyna.zurawska@polsl.pl (K.Ż.); anna.byczek-wyrostek@polsl.pl (A.B.-W.); anna.kasprzycka@polsl.pl (A.K.); 2 Department of Organic Chemistry, Bioorganic Chemistry and Biotechnology, Silesian University of Technology, Krzywoustego 4, 44-100 Gliwice, Poland

**Keywords:** 2(5*H*)-furanone, nucleophilic substitution, bioactivity, anticancer compounds, ring transformation

## Abstract

3,4-Dichloro-5-hydroxy-2(5*H*)-furanone and its dibromo analog are highly reactive molecules. Both are members of the 2(5*H*)-furanone family, which are important as pharmacophores present in drugs and natural products. Compounds possessing the 2(5*H*)-furanone skeleton isolated from plants and marine organisms exhibit bioactivity against various microorganisms and viruses and can also be used in other medical treatments. The structures of these 3,4-dihalo-2(5*H*)-furanones cause their high reactivity due to the presence of a carbonyl group on the C2 carbon conjugated with a double bond and a hydroxyl group on the C5 carbon. Two labile halogen atoms on carbons 3 and 4 offer additional possibilities for the introduction of other substituents. These structural features make 3,4-dihalo-5-hydroxy-2(5*H*)-furanones versatile reactants in chemical synthesis. In this review, we present methods of 3,4-dihalo-5-hydroxy-2(5*H*)-furanone synthesis, their applications as substrates in various chemical transformations, and examples of their biologically active derivatives.

## 1. Introduction

**Figure 1:**
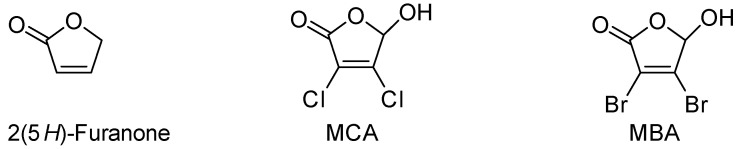
2(5*H*)-Furanone and its derivatives MCA and MBA.

3,4-Dichloro-5-hydroxy-2(5*H*)-furanone (mucochloric acid, MCA) and its bromo analog (mucobromic acid, MBA) belong to the family of 2(5*H*)-furanone derivatives, which is a wide group of organic compounds of significant pharmacological importance [1,2,3,4] (Figure 1). The 2(5*H*)-furanone ring is present in insecticides [5], fungicides [6], and antimicrobial compounds [7,8]. MCA and MBA derivatives efficiently inhibit the growth of *Salmonella typhimurium* [7] and biofilm formation by the Gram-positive bacterium *Bacillus subtilis* [8]. Experimental results suggest that this inhibition of biofilm formation and bacterial growth by these compounds is caused by their interaction with regulatory proteins [8].

A series of 2(5*H*)-furanone-based compounds synthesized from MCA exhibited antitubercular activity against *M. tuberculosis* H37Rv at a concentration of 5.6 µM/mL; they showed synergistic activity with rifampicin and additive activity with isoniazid and ethambutol [9].

**Figure 2:**
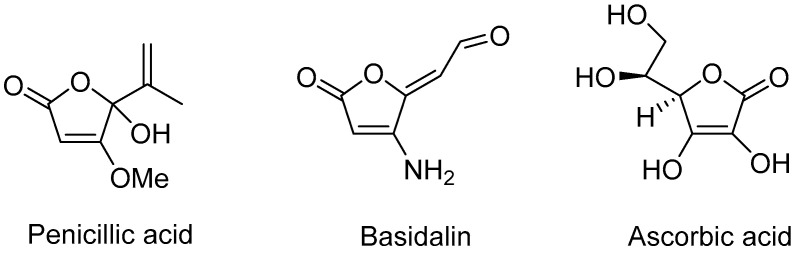
Natural metabolites containing 2(5*H*)-furanone skeleton.

**Figure 3:**
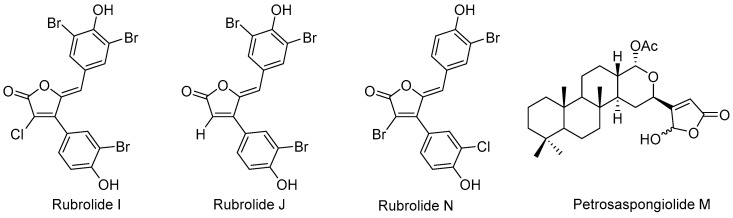
Rubrolides with anticancer activity.

The main component of the structure of MCA, 2(5*H*)-furanone, is present in secondary metabolites exhibiting specific physiological activity such as penicillic acid [10]. Basidalin is a natural metabolite isolated from *Leucoagaricus naucina* [11], while ascorbic acid (vitamin C), which is widely present in fruit and vegetables, plays an important physiological role in humans [12] (Figure 2). Metabolites isolated from the marine species *Synoicum blochmanni* and *Ritterella rubra*, known as rubrolides, show considerable activity against tumors cells [13], whereas their analogs, cadiolides, are effective inhibitors of bacterial strains [14,15] (Figure 3).

Other analogs demonstrate properties of medicinal importance, for example, petrosaspongiolide M, isolated from the Caledonian marine sponge *P. nigra*, inhibits phospholipase A2 [16]. The methanolic extracts of green onion can be applied as a chemical preventive anticancer agent [17].

## 2. Structure and Reactivity of 3,4-Dihalo-5-hydroxy-2(5*H*)-furanones

**Scheme 1:**
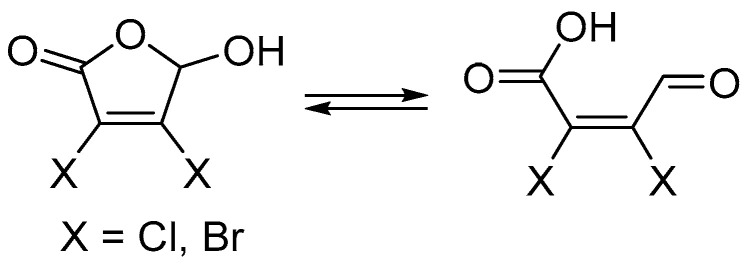
Cyclic and acyclic form of MCA and MBA.

3,4-Dihalo-5-hydroxy-2(5*H*)-furanones possess two electronegative chlorine atoms with different reactivities and a double bond conjugated with a carbonyl group, making a highly reactive molecule (Scheme 1). MCA can exist in two forms in solution: cyclic as a 3,4-dihalo-5-hydroxy-2(5*H*)-furanone and acyclic as a (*Z*)-2,3-dihalo-4-oxo-butenoic acid [18,19]. The pKa values for this equilibrium are 3.95 and 4.27 for MCA and MBA, respectively [20].

According to spectral data, in solutions at low pH, the cyclic form predominates, while under basic conditions, deprotonation leads to the acyclic form [20]. In the solid state and in organic solutions, MCA and MBA (MXA) exist only in the cyclic form [21].

**Scheme 2:**
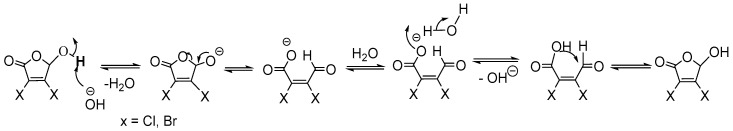
The reversibility of ring opening and cyclization in the MXA molecule.

The reversibility of ring opening and cyclization in the MXA molecule causes racemization at the C5 carbon (Scheme 2) [20]. The racemate can be separated by the formation of diastereomers with chiral alcohols such as L-menthol. This strategy comprises the synthesis of the appropriate MCA or MBA ether with L-menthol or D-borneol under acid catalysis and separation of the diastereomers [22,23].

### 2.1. Synthesis of 3,4-Dihalo-5-hydroxy-2(5H)-furanone

MCA can be obtained using various strategies. Oxidation of 3,4-dichlorofuran or 3,4-dichloro-2-furoic acid using conc. nitric acid (d = 1.42 g/mL) gave MCA in a 24% yield [24].

**Scheme 3:**
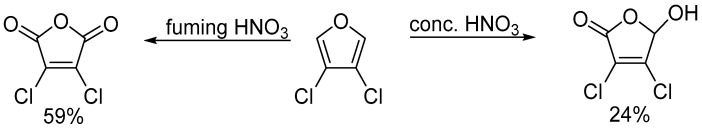
Synthesis of MCA.

**Scheme 4:**
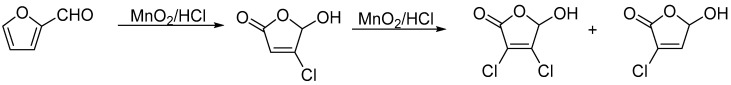
Chlorination of furfural in the presence of MnO_2_.

Fuming nitric acid oxidized 3,4-dichlorofuran to 3,4-dichloromaleic anhydride in a 59% yield (Scheme 3) [24]. Furfural is a suitable substrate for the preparation of MXA. Oxidation with manganese dioxide in the presence of hydrochloric acid led to MCA in a 59–67% yield [25,26]. A detailed investigation indicated that oxidation by MnO_2_/HCl is a twostep reaction; in the first step, 4-chloro-5-hydroxy-2(5*H*)-furanone was formed, while further heating of the reaction mixture at 60–80 °C gave MCA and a small amount of 3-chloro-5-hydroxy-2(5*H*)-furanone (Scheme 4) [25,26].

**Scheme 5:**
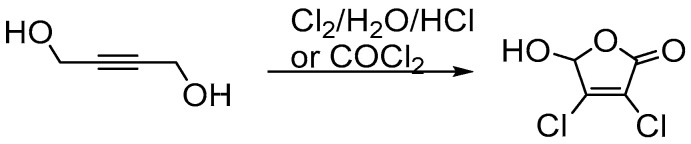
Chlorination of butyn-1,4-diol.

Butyn-1,4-diol was considered as a substrate for MCA synthesis [27]; however, the yield of product was not reported (Scheme 5).

**Scheme 6:**
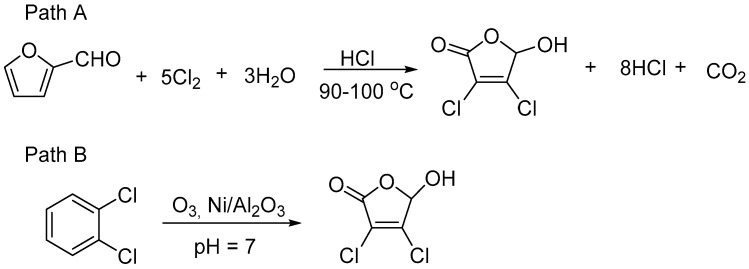
Alternative methods for MCA synthesis: oxidative chlorination of furfural with gaseous chlorine (**Path A**), and in reaction of 1,2-dichlorobenzene with ozone (**Path B**).

Oxidative chlorination of furfural with gaseous chlorine was an effective method of MCA synthesis (Scheme 6, path A) [28], with the yield of MCA exceeding 60%.

In another approach, 1,2-dichlorobenzene was oxidized with ozone in the presence of nickel-loaded metal oxides at pH 7. The yield of MCA was 89.9–100% (Scheme 6, path B) [29]. Franzen proposed another approach for the synthesis of MCA under Wittig reaction conditions [30]. The expected product was obtained in a 15% yield.

**Scheme 7:**
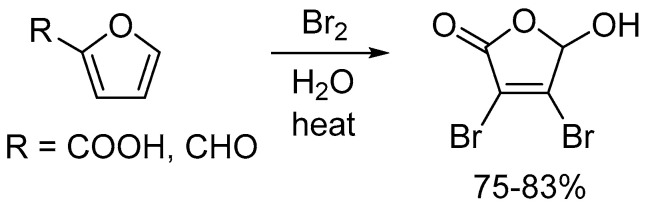
MBA synthesis.

The bromo analog of MCA, MBA, was prepared by reacting furan derivatives with bromine (Scheme 7) [31,32,33].

MBA treated with gaseous chlorine produces a product arising from the 2-bromine atom (in the open ring form, through which the reaction occurred) being replaced by a chlorine atom. The initial product was 3-bromo-2-chloro-3-formylacrylic acid, which cyclized into 4-bromo-3-chloro-2(5*H*)-furanone [34].

### 2.2. Reactivity of the 5-Hydroxyl Group

**Scheme 8:**
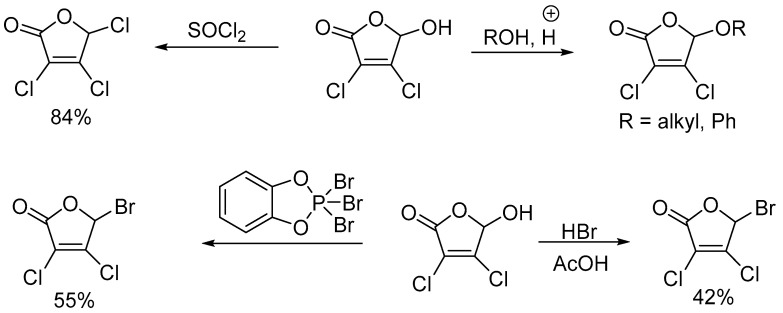
Reactivity of 5-hydroxyl group in 3,4-dihalomuco acids.

The presence of a double bond conjugated with the carbonyl group, two chlorine atoms, and a hemiacetal type of hydroxyl group makes MCA an extraordinary reactive molecule, susceptible to attack from a variety of nucleophiles. The hydroxyl group preserves properties typical of alcohols and readily forms ethers [35,36,37,38,39] and esters [40,41]. In the presence of SOCl_2_ or PBr_3_, halogenation of the hydroxyl group occurs [42,43]. Chlorination using thionyl chloride and zinc chloride as a catalyst led to 3,4,5-trichloro-2(5*H*)-furanone in a 46–52% yield [19]. Application of gaseous HBr in glacial acetic acid gave a mixture of bromination products, with the major product, 5-bromo-3,4-dichloro-2(5*H*)-furanone, being formed in 42% yield. The ratio and yield of the other two products were not reported [44]. Application of 2,2,2-tribromo-benzo-1,3,2-dioxaphosphole as a brominating agent gave the 5-bromo derivative in a 55% yield (Scheme 8) [44].

5-Alkoxy-3,4-dichloro-2(5*H*)-furanones were prepared using MCA and the appropriate alcohol. The reaction was conducted in a solution of the alcohol and in the presence of a common acidic catalyst, either sulfuric acid or *p*-toluenesulfonic acid (*p*-TSA); the yields were 70–80% [35,45].

### 2.3. Esterification of MCA

**Scheme 9:**
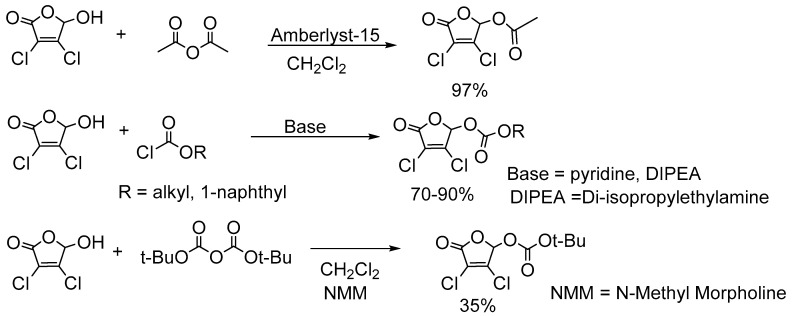
Esterification of MCA under various conditions.

5-Hydroxy-3,4-dichloro-2(5*H*)-furanone readily reacts with typical acylating compounds, such as acyl chlorides or anhydrides, in the presence of a base [41,46]. Among other acylating agents, alkyl chloroformates and di-*tert*-butyl-dicarbonate (Boc anhydride) have been used (Scheme 9) [46,47].

**Scheme 10:**
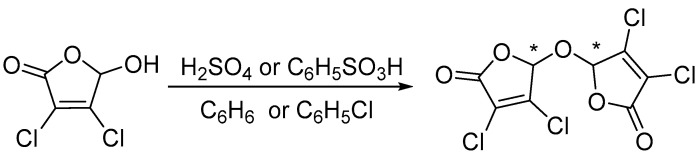
Dehydration of MCA. (*) stereogenic center.

MCA heated in a solution of benzene or chlorobenzene in the presence of a catalytic amount of sulfuric acid or benzenesulfonic acid undergoes dehydration to bis-(3,4-dichloro-5-oxo-2,5-dihydrofuran-2-yl) ether, which is isolated as a mixture of diastereomeric forms: *meso* and racemate (Scheme 10) [19,48].

### 2.4. Reactions with N-Nucleophiles

**Scheme 11:**
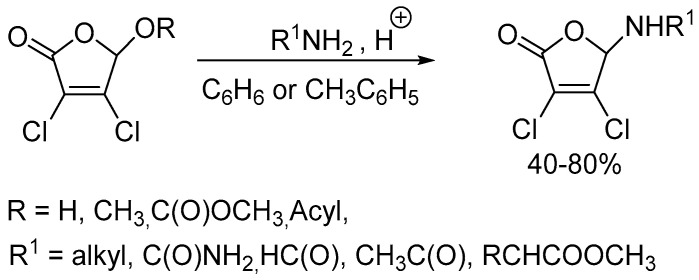
Reaction of MCA derivatives with amines in non-polar solvents.

The structure of MXA is conducive for reactions with nucleophilic reactants. Studies have particularly often examined the reactions of MXA with N-centered nucleophiles. These reactions occur easily; however, depending on the substituents on the C5 carbon in the MXA skeleton, the pH, the type of attacking N-nucleophile, and the applied solvent, the reaction gives various products. Primary and secondary aliphatic amines [5,49,50], along with amino acids [51] and amides [52,53], react with MXA in aprotic solvents, such as benzene or toluene, containing catalytic amounts of conc. sulfuric acid. The products of the reaction are the corresponding 5-aminoderivatives (Scheme 11).

**Scheme 12:**
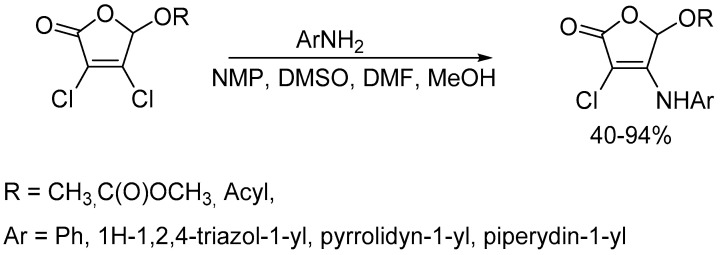
Reaction with amines in polar solvents.

Primary aryl and heteroaryl amines react with MCA in polar solvents (MeOH, NMP, DMSO, and DMF) in Michael-type addition–elimination reactions. The amino group of amines attaches to the C4 carbon of the conjugated system, and the initially formed adduct stabilizes through the elimination of hydrogen chloride. As a result of the reaction, 4-amino derivatives of MCA have been obtained in good yields (40–90%) (Scheme 12) [45,50,54,55].

**Scheme 13:**
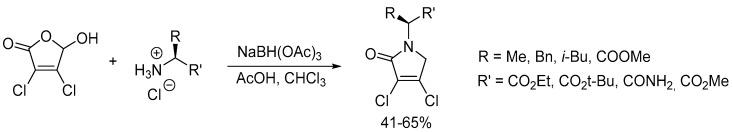
Reaction with amino acids under reducing conditions.

Treating MCA with amino acid esters under reductive conditions leads to lactams of 4-amino-2,3-dichloro-buten-2-oic acid in good yield (41–65%) (Scheme 13) [56,57,58,59].

**Scheme 14:**
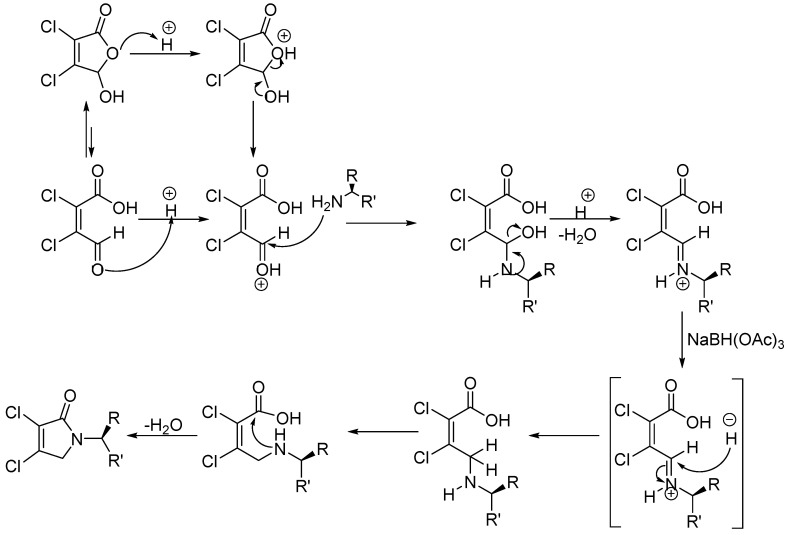
Mechanism of MCA reductive amination.

The proposed mechanism of this multistep reaction is depicted below (Scheme 14).

Under acidic conditions, the 2(5*H*)-furanone ring reacts via its acyclic form with the primary amino group of an amino acid, forming an adduct; elimination of a water molecule transforms the adduct into the corresponding imine. The reduction of the imine to a secondary amine, followed by nucleophilic attack on the carbonyl group and the elimination of a water molecule, gives the final lactam.

**Scheme 15:**
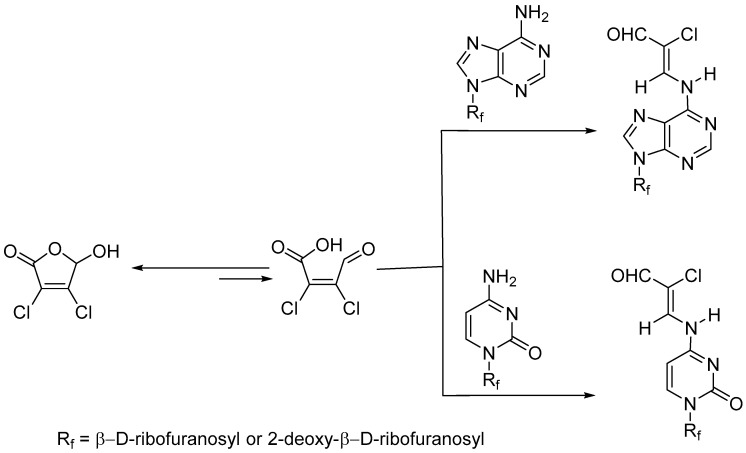
Reaction of MCA with nucleobases.

MCA and MCB react with the amino group of nucleobases, forming products of N-amination (Scheme 15) [18,60,61,62,63]. 3,4-Dihalomuco acids mostly exist in the cyclic 5-hydroxy-2(5*H*)-furanone form; however, in the presence of bases, the equilibrium between these two forms shifts to the acyclic one. The open form reacts with nucleosides containing amino groups such as adenosine and cytidine. In a reaction cascade, the alkylation of amino groups in nucleosides occurs, forming 2-chloro-2-propenal derivatives. This is a multistep reaction involving the formation of an imine by the reaction of the carbonyl group of the open MCA form with the amine group of the nucleosides, followed by chlorine atom substitution via a reaction with water, decarboxylation, and tautomerization of the formed enol into the final 2-chloro-2-propenal derivative. This N-alkenylation reaction is responsible for the cytotoxic effect of MCA and MBA [18,60,61,62,63,64,65,66,67].

**Scheme 16:**
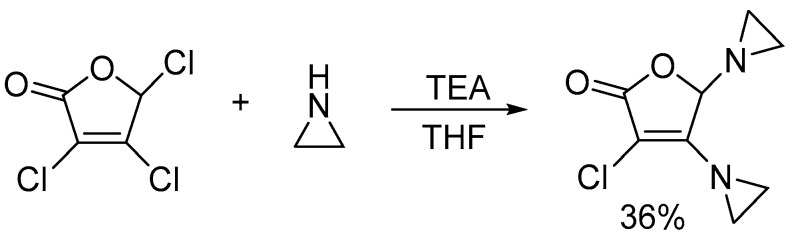
Reaction of aziridine with 3,4,5-trichloro-2(5*H*)-furanone.

Treatment of 3,4,5-trihalogeno-2(5*H*)-furanone with aziridine in the presence of TEA led to the substitution of two halogen atoms (4-Cl and 5-Cl) by this cyclic amine (Scheme 16) [68].

**Scheme 17:**
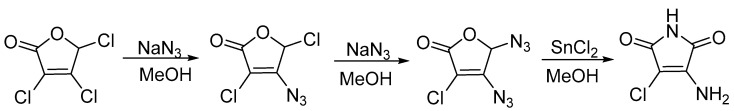
Reaction with azide and reduction of the azide groups.

3,4,5-Trichloro-2(5*H*)-furanone reacts with sodium azide in methanolic solution, giving the product of substitution at the C4 carbon. When an excess of NaN_3_ is applied, the chlorine atom on the C5 carbon is replaced by an azido group [69]. The 4,5-diazido derivative can be reduced with SnCl_2_ in a methanol solution, undergoing a transformation into 2-amino-3-chloromaleineimide (Scheme 17) [69]. 5-Alkoxy-MCA in reaction with sodium azide in methanol gave the appropriate 5-alkoxy-4-azido derivative in good yield [70,71,72].

**Scheme 18:**
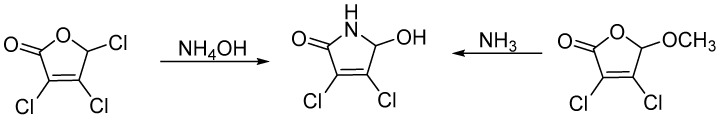
Synthesis of the 1*H*-pyrrol-2(5*H*)-one derivative.

By treating 3,4,5-trichloro-2(5H)-furanone with ammonium hydroxide or 3,4-dichloro-5-methoxy-2-(5*H*)-furanone with ammonia, Ratts and Philips accomplished the synthesis of 3,4-dichloro-5-hydroxy-1*H*-pyrrol-2(5*H*)-one (Scheme 18) [43].

**Scheme 19:**
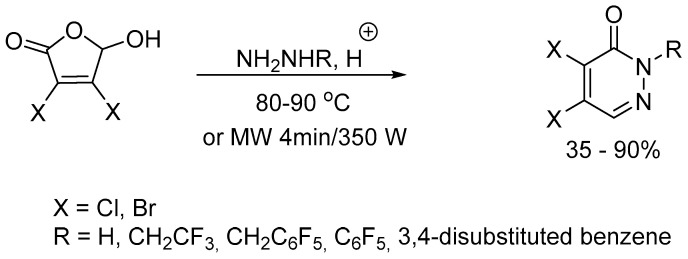
Reaction of MXA with bifunctional N-nucleophiles.

The reaction of MCA or its 5-aryl derivative with hydrazine or its derivatives in aqueous acidic solutions at elevated temperature leads to 2(5*H*)-furanone ring transformation. The appropriate 4,5-dihalogeno-3(2*H*)-pyridazinone can be prepared in satisfactory yield (35–90%) (Scheme 19) [37,58].

**Scheme 20:**
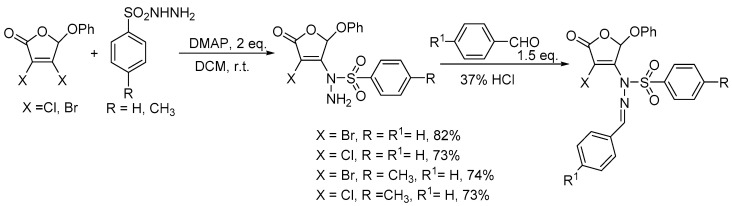
Reaction with arylsulfonyl hydrazines.

Methoxy or phenoxy ethers of 3,4-dihalogeno-2-(5*H*)-furanone reacted with arylsulfonyl hydrazines, forming products from 4Br atom substitution, which condensed with substituted benzaldehydes and were transformed into the appropriate (*E*)-hydrazones. The cytotoxic activities against three human cancer cell lines (MCF-7, U87, and A549) of the obtained hydrazones were evaluated by an MTT assay, which indicated moderate cytotoxicity, with the best IC_50_ value being 14.35 μM (Scheme 20) [73].

### 2.5. Reactions with O-Nucleophiles

**Scheme 21:**
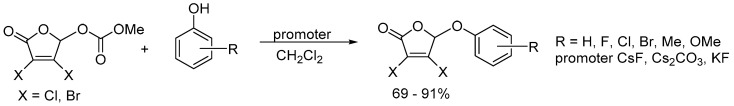
Synthesis of 5-phenoxy derivatives.

Phenol and its derivatives are efficient nucleophiles in their reaction with 3,4-dihalogeno-2(5*H*)-furanone derivatives. The 5-methyl carbonates of MCA and MCB react with substituted phenols in the presence of a promoter in methylene chloride solution. The expected products of carbonate group substitution, the 5-phenoxy derivatives, were isolated in high yield (Scheme 21) [41].

**Scheme 22:**
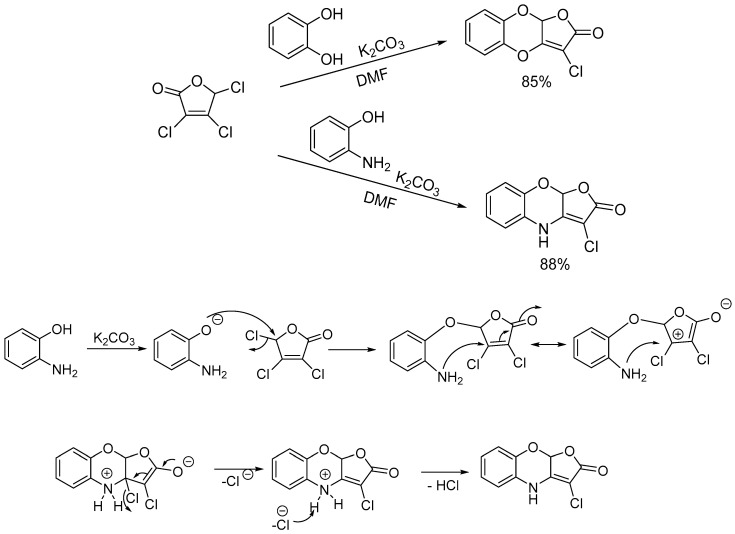
Reaction of MCA with *o*-substituted phenols.

Pyrocatechol and 2-aminophenol react with 3,4,5-trichloro-MCA in the presence of K_2_CO_3_, forming tricyclic derivatives in high yield. In the case of 2-aminophenol, the only isolated product was the 1,4-oxazine derivative (Scheme 22) [74]. Based on experimental data and NMR analysis, the formation of the 1,4-oxazine derivative occurs by an initial attack of the phenoxy oxygen atom on the harder chlorine atom joined to the C5 carbon, followed by a Michael-type addition of the amino group to the conjugated system of the carbonyl group-double bond, with subsequent elimination of a hydrogen chloride molecule (Scheme 22).

### 2.6. Reaction with S-Nucleophiles

**Scheme 23:**
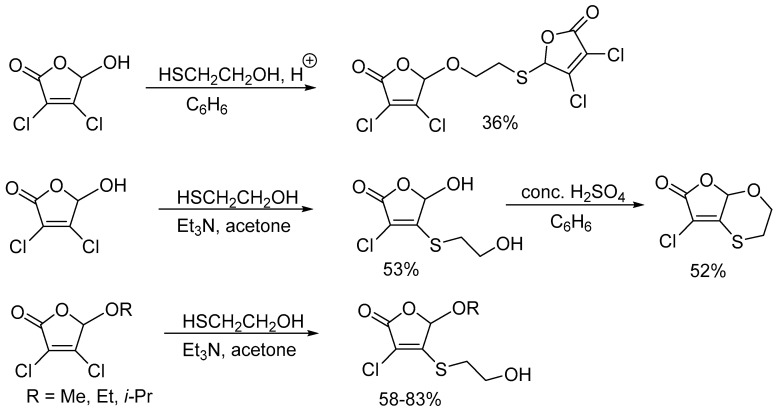
Reaction of MCA with sulfur derivatives.

**Scheme 24:**
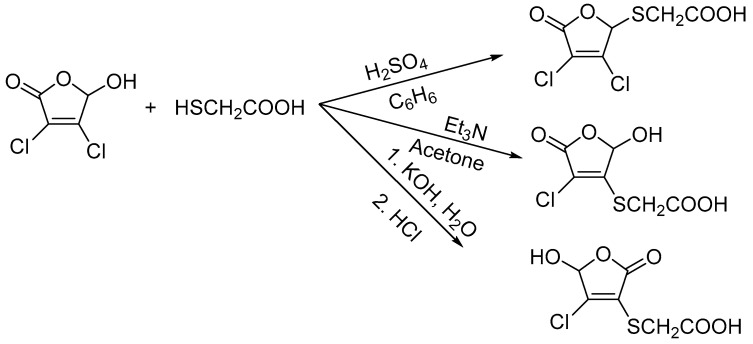
Reaction of MCA with thioglycolic acid.

MCA and 5-alkoxy-3,4-dichloro-2(5*H*)-furanone are susceptible to nucleophilic attack by mercaptans [75,76,77]. Depending on the applied conditions, substitution occurs on the C4 or C5 carbon of the MCA molecule (Scheme 23). MCA treated with 2-mercaptoethanol in benzene in the presence of a catalytic amount of H_2_SO_4_ forms a molecule containing two 3,4-dichloro-2(5*H*)-furanone units, namely 3,4-dichloro-5-[2-(3,4-dichloro-5-oxo-2,5-dihydrofuran-2-ylsulphanyl)ethoxy]-2(5*H*)-furanone [75]. When MCA reacts with 2-mercaptoethanol in the presence of triethylamine in acetone solution, the product of substitution on carbon C4 is formed. The latter undergoes a condensation catalyzed by sulfuric acid, giving 7-chloro-2,3,4a,6-tetrahydrofuro[2,3-b][1,4]oxathiin-6-one in a 52% yield, the compound being formed as a result of 2-mercaptoethanol’s bifunctional character. 5-Alkoxy-3,4-dichloro-2(5*H*)-furanone, under treatment with 2-mercaptoethanol under basic conditions, gave the product of 4-chlorine atom substitution. Similarly, in the reaction of MCA with mercaptoacetic acid (thioglycolic acid), depending on the applied conditions, the products of substitution on carbon C5, C4, or C3 were obtained (Scheme 24) [76].

**Scheme 25:**
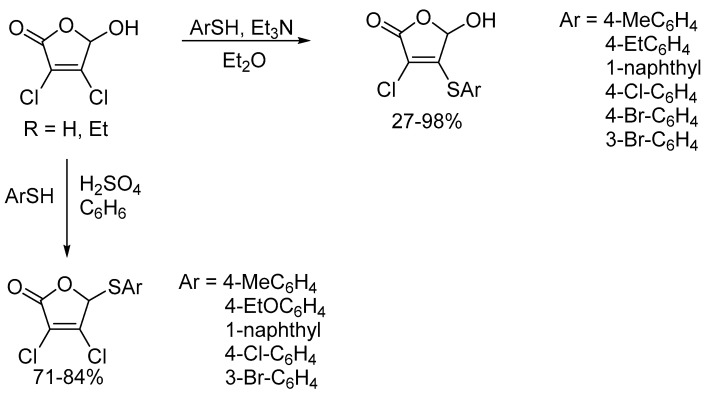
Synthesis of 4- or 5-thioaryl-3,4-dihalo-mucoacids.

MCA and MBA react readily with other sulfur derivatives such as glutathione. The reaction occurs at the C4 carbon by substitution of the chlorine atom [77]. MCA readily reacts with thiophenol derivatives [78,79,80]; treatment of MCA with thiophenols under acidic conditions in benzene gave 5-thioaryl derivatives in high yields. In the presence of triethylamine in diethyl ether solution, the reaction between MCA and thiophenols occurs according to the addition–elimination mechanism, giving appropriate products in 27–98% yields (Scheme 25) [80].

**Scheme 26:**
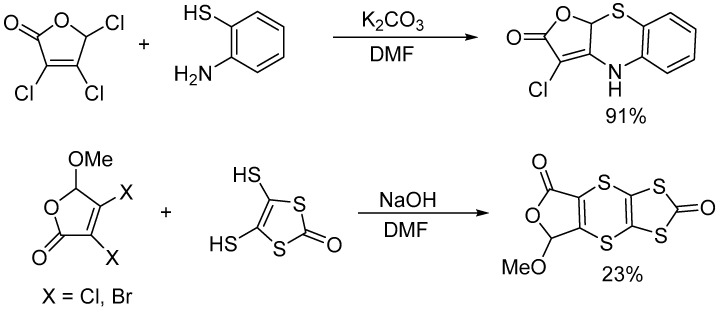
Synthesis of tricyclic products.

3,4,5-Trichloro-2(5*H*)-furanone reacts with 2-aminothiophenol under the same conditions as 2-aminophenol. The corresponding tricyclic derivative was obtained in exceptionally good yield (91%) [74]. 5-Methoxy-3,4-dihalogeno-2(5*H*)-furanone treated with 2-oxo-1,3-dithio-4,5-dithiolate formed the appropriate tricyclic derivative (Scheme 26) [81].

**Scheme 27:**
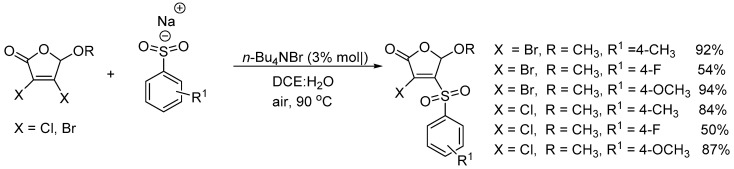
Preparation of 4-sulfonylated derivatives.

5-Alkoxy-3,4-dihalo-2(5*H*)-furanones are easily sulfonylated on the C4 carbon by sodium benzenesulfinates. The reaction occurs under phase-transfer catalysis (PTC) conditions in a 1,2-dichloroethane: water system (DCE:H_2_O, 5:1) via a metal-free radical pathway. The reaction has been accomplished at a scale of up to 10 g (Scheme 27) [82].

**Scheme 28:**
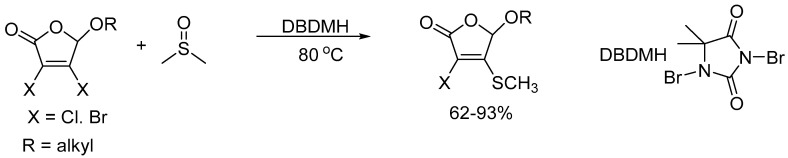
Synthesis of MXA 4-methylthio ethers.

Treatment of 5-alkoxy 3,4-dihalo-2(5H)-furanones with DMSO under reducing conditions, in the presence of DBDMH (1,3-dibromo-5,5-dimethylhydantoin); the series of appropriate 4-thiomethyl ethers was obtained in good yield (Scheme 28) [83].

### 2.7. Reaction with Se-Nucleophiles

**Scheme 29:**
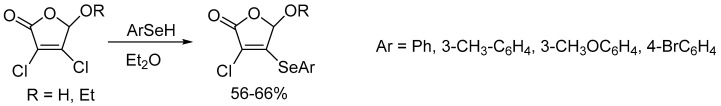
Synthesis of selenium derivatives.

Reactive molecules of MCA can react with other nucleophiles with an easily shared electron pair. Selenophenols in diethyl ether solution easily displace the 4-chlorine atom in the MCA 5-ethoxy derivative. The reaction products were isolated in particularly good yield (Scheme 29) [84].

**Scheme 30:**
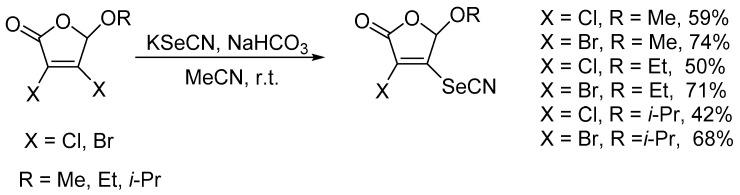
Synthesis of 5-alkoxy-3-halo-4-selenocyanato-2(5*H*)-furanones.

5-Alkoxy-3,4-dihalo-2(5*H*)-furanones react with potassium selenocyanate (KSeCN) in acetonitrile under the presence of basic conditions. The products of substitution on carbon C4 of the 2(5H)-furanone ring were obtained in satisfactory yield (Scheme 30) [85].

### 2.8. Reactions with Phosphorus Derivatives

**Scheme 31:**
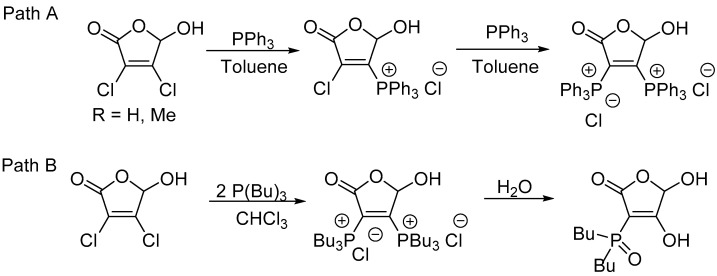
Reaction of MCA with triphenylphosphine (**Path A**) and the reaction of MCA with tributylphosphine (**Path B**).

The phosphorus atom, with its unshared electron pair in phosphines and phosphites, is an efficient nucleophile in reaction with MCA. Triphenylphosphine and trialkylphosphines react with MCA to form the corresponding phosphonium salts, a product of chlorine atom substitution at the C4 carbon. When a phosphine is used in excess, both chlorine atoms undergo substitution (Scheme 31) [86].

**Scheme 32:**
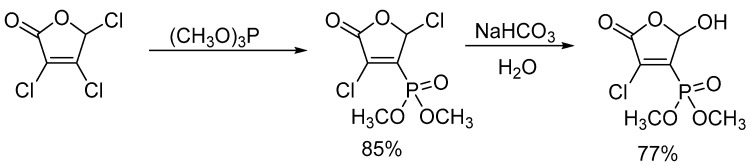
Synthesis of MCA phosphonate.

Unsubstituted 3,4-dichloro-2(5*H*)-furanone reacts with tributylphosphine, forming a double tributylphosphonium salt, which, in the presence of water, converts into the corresponding phosphine oxide (Scheme 31). Trimethoxyphosphite treated with 3,4,5-trichloro-2(5*H*)-furanone or 5-methoxy-3,4-dichloro-2(5*H*)-furanone reacts according to an addition–elimination mechanism, creating a phosphonate that is the product of substitution at the C4 carbon of the 2(5*H*)-furanone ring, which under basic conditions is transformed into another phosphonate that has an OH group at C5 (Scheme 32) [43].

### 2.9. C-C Bond Formation

**Scheme 33:**
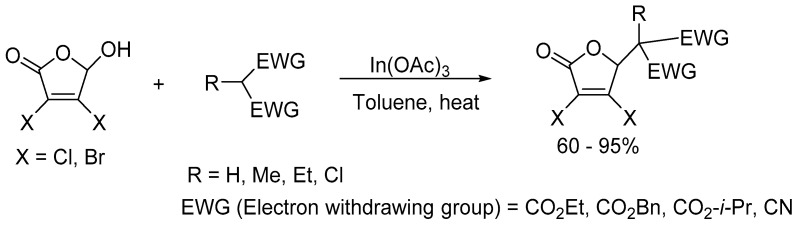
Knoevenagel reaction of MXA substrates.

3,4-Dibromo and 3,4-dichloro-2(5*H*)-furanones react at the C5 carbon with compounds having an active hydrogen atom in the presence of Lewis acid (e.g., In(OAc)_3_) in good yields (60–95%). This reaction is classified as a Knoevenagel condensation (Scheme 33) [87].

**Scheme 34:**
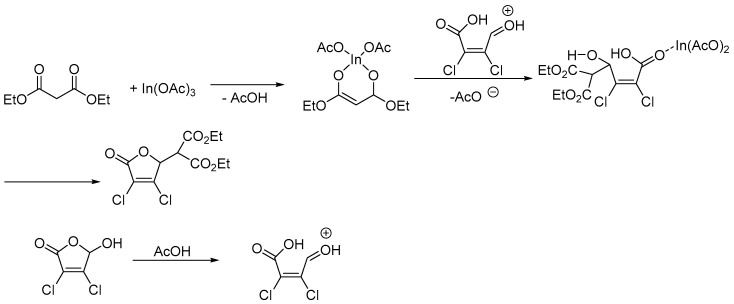
Mechanism of Knoevenagel condensation with the In(OAc)_3_ catalyst.

The postulated mechanism involves the ring opening of MCA in the presence of acetic acid released from indium triacetate during the reaction with the Knoevenagel substrate (Scheme 34) [87].

**Scheme 35:**
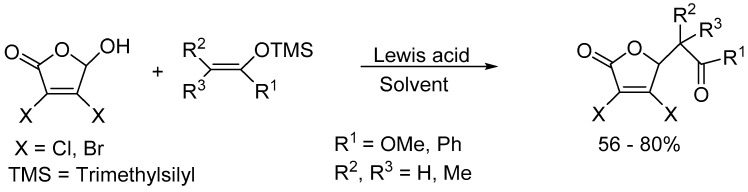
Mukaiyama aldol reaction.

Another strategy for C5 alkylation of 3,4-dihalogeno-5-hydroxy-2(5*H*)-furanones is the reaction with silylated enol ethers catalyzed by Lewis acids (Mukaiyama aldol reaction). The reaction occurs in solvents of different polarity: toluene, methylene chloride, THF, diethyl ether, and nitromethane. Higher yields of products were achieved when zinc chloride or scandium triflate in diethyl ether or nitromethane solutions were used. The reaction begins at a decreased temperature (−20 °C) during an initial period, and then the temperature is allowed to rise to ambient (Scheme 35) [88,89].

**Scheme 36:**
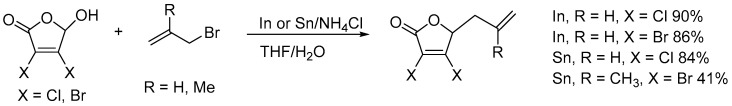
Barbier reaction of MXA with allyl derivatives.

Alkylation of 3,4-dihalogeno-5-hydroxy-2(5*H*)-furanones on the C5 carbon can be achieved by a Barbier-type reaction, where allyl bromides are used as the alkylating agents in the presence of a tin or indium catalyst, with a mixture of water and either THF or methanol being used as the solvent. The yield of alkylation products varied over a range of 41–90% (Scheme 36) [90].

**Scheme 37:**
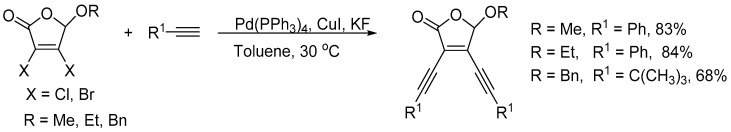
Sonogashira coupling reaction.

5-Alkoxy and 5-acetoxy derivatives of dihalomuco acids react with monosubstituted acetylene derivatives in the presence of a palladium catalyst and KF as the promoter (Sonogashira coupling reaction). Under the applied conditions, both halogen atoms are exchanged. The yield of products varied over a range of 68–84% (Scheme 37) [91].

### 2.10. Arylation of MCA

**Scheme 38:**
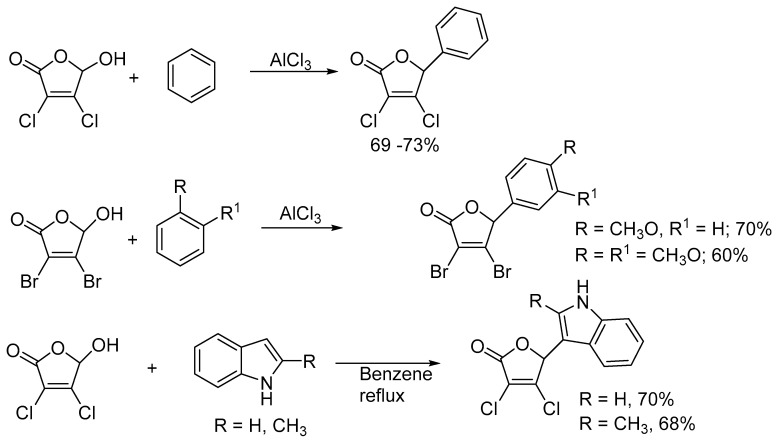
Arylation of MCA.

3,4-Dihalogeno-5-hydroxy-2(5*H*)-furanone in the presence of Lewis or Brönsted acids reacts with arenes and heteroarenes, forming a new bond between the C5 carbon of the 2(5*H*)-furanone and the aromatic compound. Formally, the reaction can be considered as an aromatic electrophilic substitution of the arene. The reaction was successful with methoxybenzene, 1,3,5-trimethoxybenzene, indole, and 2-methylindole, with the yields of the arylation products being satisfactory (60–73%) (Scheme 38) [37,92,93,94,95].

**Scheme 39:**
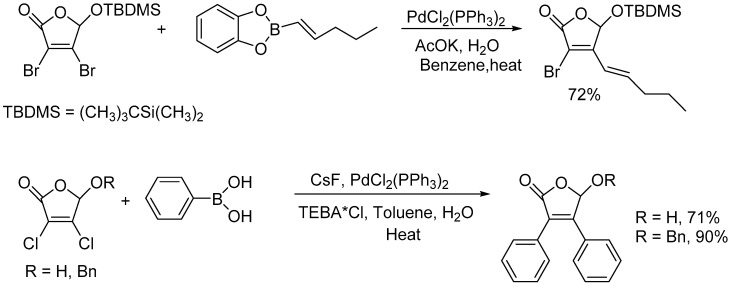
Suzuki coupling reaction.

A new C-C bond to the MCA molecule and its bromo counterpart can be formed by a reaction involving organometallic catalysts. Thus, 3,4-dibromo-5-*O*-*tert*-butyldimethylsilyl-2(5*H*)-furanone treated with 2-(penten-1-yl)benzo[*d*][1,2,3]dioxaborole in the presence of a palladium catalyst and potassium acetate gave the product of C4 alkylation in a 72% yield by means of a Suzuki reaction [96]. MCA reacts with phenylboronic acid under PTC conditions in a benzene:water mixture (1:1) as a reaction medium and benzyltriethylammonium chloride (TEBA*Cl) as a PTC catalyst. Under these conditions, two phenyl groups were introduced at the C3 and C4 carbons of the 2(5*H*)-furanone ring in satisfactory yield (Scheme 39) [96,97,98].

### 2.11. Wittig Reaction

**Scheme 40:**
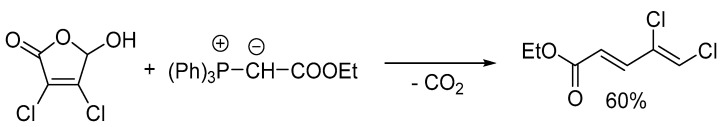
Reaction of MCA with phosphorus ylide.

MCA reacts with phosphorus ylides, forming a double bond. This reaction confirms that the ring open form of MCA was present, allowing the carbonyl group to form a double bond. The formation of the double bond is associated with decarboxylation (Scheme 40) [99].

### 2.12. Reduction of MXA

**Scheme 41:**
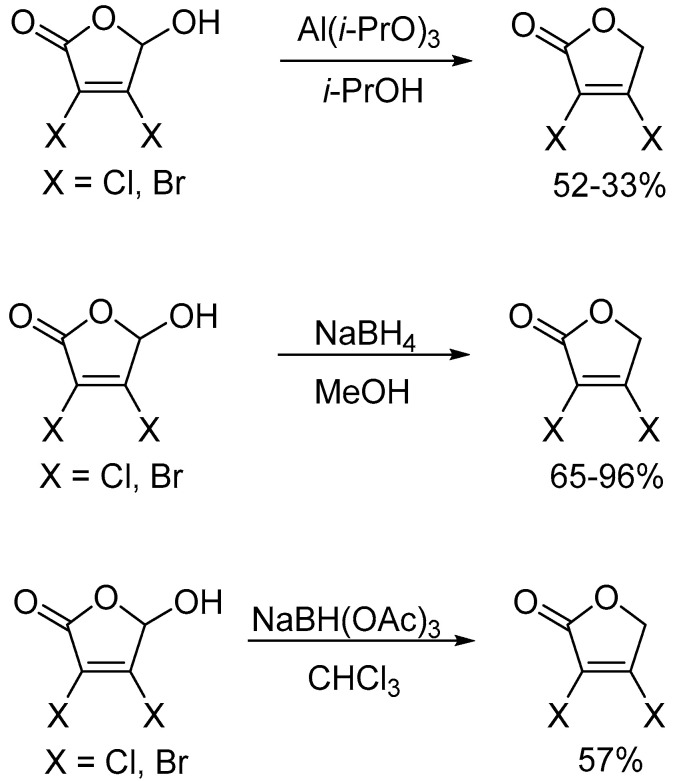
Reduction of mucohalic acids.

**Scheme 42:**
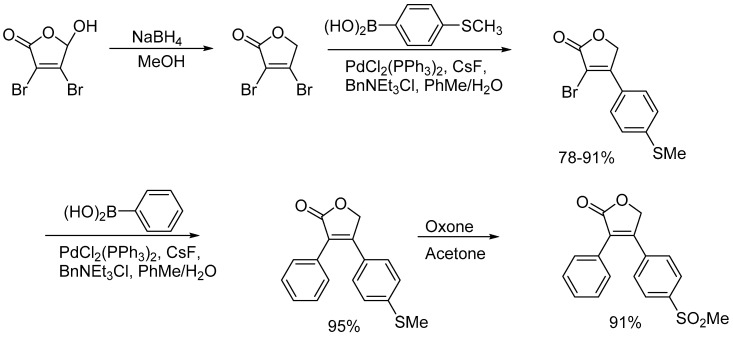
Synthesis of 3,4-diaryl-2(5*H*)-furanone via 3,4-dibromo-2(5*H*)-furanone.

3,4-Dihalogeno-5-hydroxy-2(5*H*)-furanones transform into γ-lactones with yields varying over a range of 33–96% under treatment with common reducing agents, aluminum isopropoxide, sodium borohydride, and sodium triacetoxyborohydride having been used (Scheme 41) [68,97,98,100]. The γ-lactones are important and versatile substrates in the synthesis of compounds of high importance used as pharmaceuticals [3]. They are used in the synthesis of rubrolides, the preparation of chiral γ-substituted butyrolactones, and the formation of amino alcohols [97,101,102]. Dihalomuco acids (MCA or MBA) were applied in the synthesis of rofecoxib (Vioxx), a COX-2 selective, nonsteroidal anti-inflammatory agent introduced to the market in 1999 as a drug against rheumatoid arthritis and acute pain. In 2004, it was withdrawn from the market due to a serious risk of heart attack associated with long-term, high-dosage use. Scheme 42 depicts the synthesis of rofecoxib via the bis-arylation of 3,4-dibromo-2(5*H*)-furanone using Suzuki coupling reactions. Treatment of 3,4-dibromo-α,β-unsaturated-γ-butyrolactone with 4-(methylsulfanyl)phenylboronic acid in a two-phase solvent system (water: toluene) in the presence of a palladium salt and PTC catalyst led to a 4-aryl-3-halo derivative in high yield. In the next step, performed under the same reaction conditions, the second aryl group was introduced. Oxidation of the sulfide group by Oxone in a wet acetone solution gave the desired product in a 74% yield [97]. Similar results were obtained when 3,4-dichloro-α,β-unsaturated-γ-butyrolactone was used as a substrate.

**Scheme 43:**
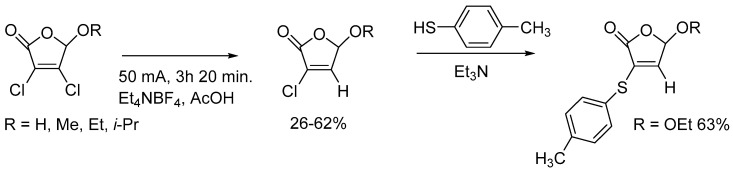
Electrochemical reduction of the MCA derivative.

5-Alkoxy derivatives of dihalomuco acids in acetonitrile were reduced on lead electrodes to 5-alkoxy-3-chloro-2(5*H*)-furanones. The reduction occurred in the presence of acetic acid as a proton source (Scheme 43) [103].

The position of chlorine replacement by hydrogen in the 2(5*H*)-furanone ring was confirmed by ^1^H NMR analysis and the reaction with *p*-thiocresol [103].

### 2.13. Reactions Involving the Open Form of MCA

**Scheme 44:**
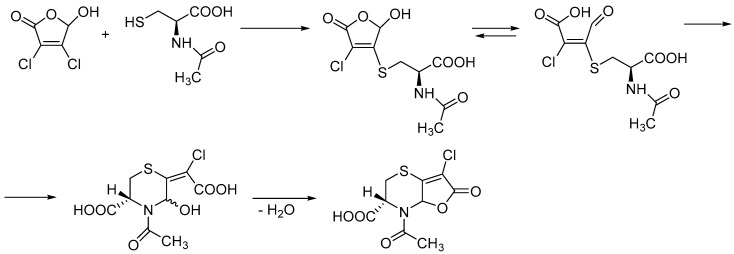
Transformation of the MCA molecule under treatment with N-acetylcysteine.

3,4-Dihalomuco acids mostly exist in the cyclic 5-hydroxy-2(5*H*)-furanone form; however, in the presence of bases, the equilibrium between these two forms shifts to the acyclic one. The open form reacts with the amino groups of nucleobases (Scheme 15). A similar mechanism is possible for amino acids present in living systems. A model study using N-acetylcysteine indicated that in its reaction with MCA, the open ring form is involved (Scheme 44) [104]. An initial nucleophilic attack of cysteine on MCA gives the product of substitution at the C4 carbon. In the next step, the 2(5*H*)-furanone ring opens, and the aldehyde group reacts with the amino group of cysteine, forming a cyclic product, which is responsible for the mutagenic properties of MCA with *Salmonella typhimurium* (TA100). It is believed that the formation of this cyclic product and the N-alkenylation of nucleobases are responsible for the cytotoxic effect of MCA and MBA [18,60,61,62,63,64,65,66,67].

### 2.14. Bioactivity of 3,4-Dihalogeno-5-hydroxy-2(5H)-furanones

The presence of the 2(5*H*)-furanone skeleton in compounds of natural origin exhibiting bioactivity against various biotargets makes them among the leading structures for research laboratories seeking new compounds with better activity and selectivity. The 2(5*H*)-furanone scaffold is present in diverse compounds exhibiting a wide range of activity: anticancer, antibacterial, antifungal, and antiviral. 3,4-Dihalogeno-2(5*H*)-furanone derivatives exhibit various anticancer activities inhibiting key enzymes such as kinases, COX-1, topoisomerase I, or p53 activity. The MCA and MBA derivatives can block the cell cycle phases.

**Figure 4:**
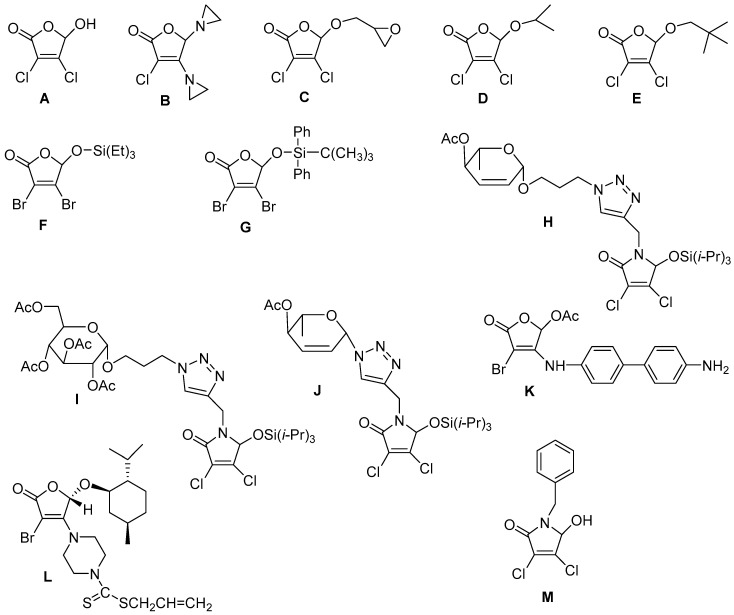
MXA derivatives exhibiting activity against tumor cells.

**Figure 5:**
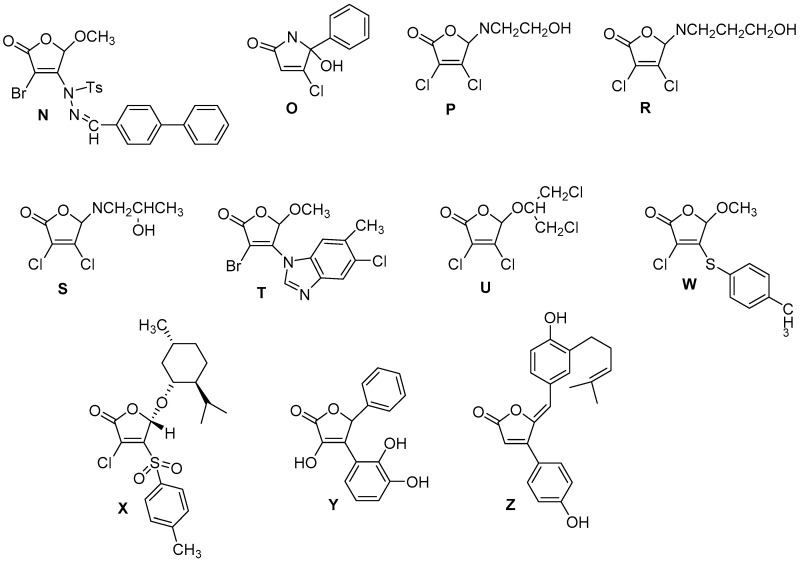
MXA derivatives exhibit diverse types of bioactivities.

Examples of bioactive 3,4-dihalomuco acids are depicted in Figure 4 and Figure 5. MCA **A** exhibited moderate cytotoxic activity against BEAS-2B healthy cell lines isolated from normal human bronchial epithelium (45.0 ± 1.3 µM). IC_50_ (50% inhibitory concentration) values of 50 nM and 30 nM were determined for the aziridine **B** and epoxide **C**, respectively, in the murine colon adenocarcinoma (MAC13, MAC16) cell lines [68]. 5-Alkoxy-3,4-dichloro-2(5*H*)-furanones **D** and **E** exhibited high selectivity toward A549 cancer cells with IC_50_ 6.7 µg and 7.7 µg, respectively. Further investigation revealed that **D** and **E** led to G2 phase cell cycle arrest and the induction of caspase-independent cell death [45]. The silyl ethers of MBA, **F** and **G**, exerted strong antiproliferative activity against HCT116 colon cancer cells, inducing apoptosis. A detailed investigation of the mechanism of action revealed that this activity stems from the downregulation of survivin and the activation of caspase-3 [39]. The newly reported derivatives **H**, **I**, and **J** caused a significant decrease in the cell viability of the HCT116 and MCF-7 cell lines [105]. The compound **K** exhibited significant inhibitory activity against MCF-7 human breast cancer cells with an IC_50_ value of 11.8µM and low toxicity toward HaCaT human normal cells (>100 µM), respectively [54].

Dithiocarbamate **L**, containing a 2(5*H*)-furanone-piperazine group, showed good in vitro cytotoxic activity against the HeLa and SMMC-7721 cell lines with an IC_50_ of 0.06 ± 0.01 µM and 0.006 ± 0.04 µM for 72 h, respectively [23]. As a result of the reductive amination reaction of 3,4-dihalogeno-2(5*H*)-furanone derivatives in the presence of various amines, highly functionalized α,β-unsaturated α-halogeno-β-aryl-γ-butyrolactams were obtained [4,97]. Pyrrole analogs **M** represent a promising class of small molecules selectively enhancing by up to 2000-fold oncolytic virus (OV) replication in cancer tissue. OVs are considered as potent tools in anticancer therapy [106,107].

The circular supercoiled DNA of bacteriophage plasmid SC-ΦX174 under treatment with MCA converts to relaxed (*R*) and linear (*L*) forms [68]. Compounds based on the MBA scaffold **N** showed cytotoxic activity comparable to cisplatin against MCF-7 cells with an IC_50_ 14.35 µM and 15.27 µM, respectively (Figure 5) [73]. 5-Arylated-5-hydroxy-pyrrol-2-ones, **O**, prepared from MCA, were investigated as CCK selective ligands. The CCK binding affinity expressed in IC_50_ using cortex and pancreatic membranes was established as 8 nM [108].

Introduction of a 5-(ω-hydroxyalkylamino) substituent into the 2(5*H*)-furanone skeleton gave compounds that exhibited a high inhibition of growth in the *T*.*b. rhodesiense* and *T. cruzi* parasites. Compounds **P**–**S** totally inhibited the growth of *T. cruzi* at a concentration of 4.8 µg/mL, which is only four times higher than a standard dose of Benznidazole^®^ [109].

Derivatives of MBA and MCA are active against diverse types of bacteria [110,111,112,113,114]. The 3-bromo-4-benzimidazolyl-5-methoxy-2(5*H*)-furanone **T** showed an MIC of 8 µg/mL against multiresistant *Staphylococcus aureus* (MRSA) [8,110]. Compounds **U** and **W** bearing an alkoxy substituent on the C5 carbon were effective inhibitors of growth and biofilm formation by *Bacillus subtilis* [8]. Compound **X** exhibited minimal inhibitory and bactericidal concentration values (MIC and MBC) against biofilm-embedded *S. aureus* equal to 10 mg/L and 40 mg/L, respectively [114].

4,5-Diaryl-3-hydroxyfuranones exhibit anti-inflammatory and antioxidant activities [115,116]. Compound **Y** was the most powerful scavenger of free radicals such as the DPPH (2,2-diphenyl-1-picrylhydrazyl) radical with IC_50_ = 10.3 µM [115]. Rubrolide **Z**, isolated from the fermentation broth of the fungus *Aspergillus terreus* OUCMDZ-1925, showed comparable antioxidation activity against 2,2′-azino-di(3-ethylbenzothiazoline-6-sulfonic acid) (ABTS) radicals to those of Trolox (6-hydroxy-2,5,7,8-tetramethylchroman-2-carboxylic acid) and ascorbic acid with an IC_50_ value of 1.33 mM (Figure 5) [116].

## 3. Conclusions

3,4-Dihalo-5-hydroxy-2(5*H*)-furanones are versatile reactants for organic synthesis. Their unique structure makes diverse structural changes of their skeleton possible, with the range of potential reactants being extremely high. The availability of substrates and their confirmed bioactivity against various biotargets has led to continuous interest in seeking new derivatives and creating MCA- and MBA-derived leading structures. It should be mentioned that unmodified MCA and MBA are direct genotoxins and possible carcinogens. Both MCA and MBA alkylate nucleobases contain amino groups (cytosine, adenine, and guanine) and disrupt the cellular functions of nucleic acids. Novel modifications of 3,4-dihalo-5-hydroxy-2(5*H*)-furanone are still possible, especially in strategies involving the creation of C-C bonds between functionalized units. In the future, research should focus on the synthesis of 5-substituted derivatives containing chiral centers that are different from simple terpenes. Introduction of a chiral substituent on the C5 carbon of 2(5*H*)-furanone prevents possible racemization, enabling diastereomer separation, and can thus create compounds with new biological activities.

## Data Availability

Not applicable.
